# The genome sequence of Bastard Balm, *Melittis melissophyllum* L. (Lamiales: Lamiaceae)

**DOI:** 10.12688/wellcomeopenres.27850.1

**Published:** 2026-09-18

**Authors:** Maarten J. M. Christenhusz, Sahr Mian, Ilia J. Leitch, Royal Botanic Gardens Kew Genome Acquisition Lab, Plant Genome Sizing Collective, Darwin Tree of Life Barcoding collective, Wellcome Sanger Institute Tree of Life Management, Samples and Laboratory team, Wellcome Sanger Institute Scientific Operations: Sequencing Operations, Wellcome Sanger Institute Tree of Life Core Informatics team, Tree of Life Core Informatics collective, Darwin Tree of Life Consortium

**Affiliations:** 1 Royal Botanic Gardens Kew, Richmond, Surrey, England, UK; 2 Botanical Society of Britain and Ireland, St Albans, Hertfordshire, England, UK

**Keywords:** Melittis melissophyllum, Bastard Balm, genome sequence, chromosomal, Lamiales

## Abstract

We present a genome assembly of *Melittis melissophyllum* (Bastard Balm; Streptophyta; Magnoliopsida; Lamiales; Lamiaceae). The primary assembly has a total length of 436.70 megabases. Most of the primary assembly (97.71%) is scaffolded into 17 chromosomal pseudomolecules. The mitochondrial genome assembly has a length of 366.69 kilobases and the plastid genome assembly has a length of 153.23 kilobases. This assembly was generated as part of the Darwin Tree of Life project, which produces reference genomes for eukaryotic species found in Britain and Ireland.

## Species taxonomy

Eukaryota; Viridiplantae; Streptophyta; Streptophytina; Embryophyta; Tracheophyta; Euphyllophyta; Spermatophyta; Magnoliopsida; Mesangiospermae; eudicotyledons; Gunneridae; Pentapetalae; asterids; lamiids; Lamiales; Lamiaceae; Lamioideae; Stachydeae; *Melittis*; *Melittis melissophyllum* L. (NCBI:txid457147)

## Background

We sequenced the genome of *Melittis melissophyllum* as part of the Darwin Tree of Life Project. The sequenced specimen was grown from seed at the Royal Botanic Gardens, Kew that was originally collected from Neyland, Westfield Pill, Pembrokeshire, Wales, United Kingdom.

*Melittis melissophyllum* (Figure 1) is a clump-forming, long-lived perennial herb with opposite leaves and axillary flowers. The pink to white, fused corolla is four-lobed with the lower lobe largest and marked inside with purple. The four stamens are in two opposite pairs and inserted in the corolla tube under the upper, hooded lobe. The name *Melittis* is derived from the Greek for bee, which is one of the insects recorded pollinating the nectar-rich flowers. *Melittis melissophyllum* is the only species in the genus, with the epithet meaning, “with leaves like *Melissa*” – the lemon balm *Melissa* L. being another genus within the family Lamiaceae.

Native to Europe and Anatolia ( POWO, 2026), Bastard Balm is sometimes cultivated as a garden ornamental. The species is variable in colour across its range and different colour forms exist, from pure white to dark pink. In Britain it is found in woodland, woodland margins, clearings, hedge banks and river valley scrub on damp base-rich soils in Cornwall, Devon, the New Forest and Pembrokeshire. It is absent from Ireland ( Walker, 2023). The plant is sensitive to grazing and does not like competition. Indeed, the lack of coppicing and increased grazing from deer and horses may contribute to the reported local declines in the New Forest and the south-west ( Walker *et al.*, 2017).

The sporophytic chromosome count most frequently reported for this species, including material collected from Britain, is 2 *n* = 30 (e.g. Henniges *et al.* , 2022; Morton, 1973). Some variation has nevertheless been reported, including a count of 2 *n* = 36 from Portugal ( Fernandes & Leitão, 1984). The relationship between these published chromosome counts and the 17 chromosomal pseudomolecules in this assembly remains unresolved.

We present a chromosome-level genome sequence of *Melittis melissophyllum* which is the first reported for this species ( Schwacke *et al.*, 2025).

**Figure 1.**
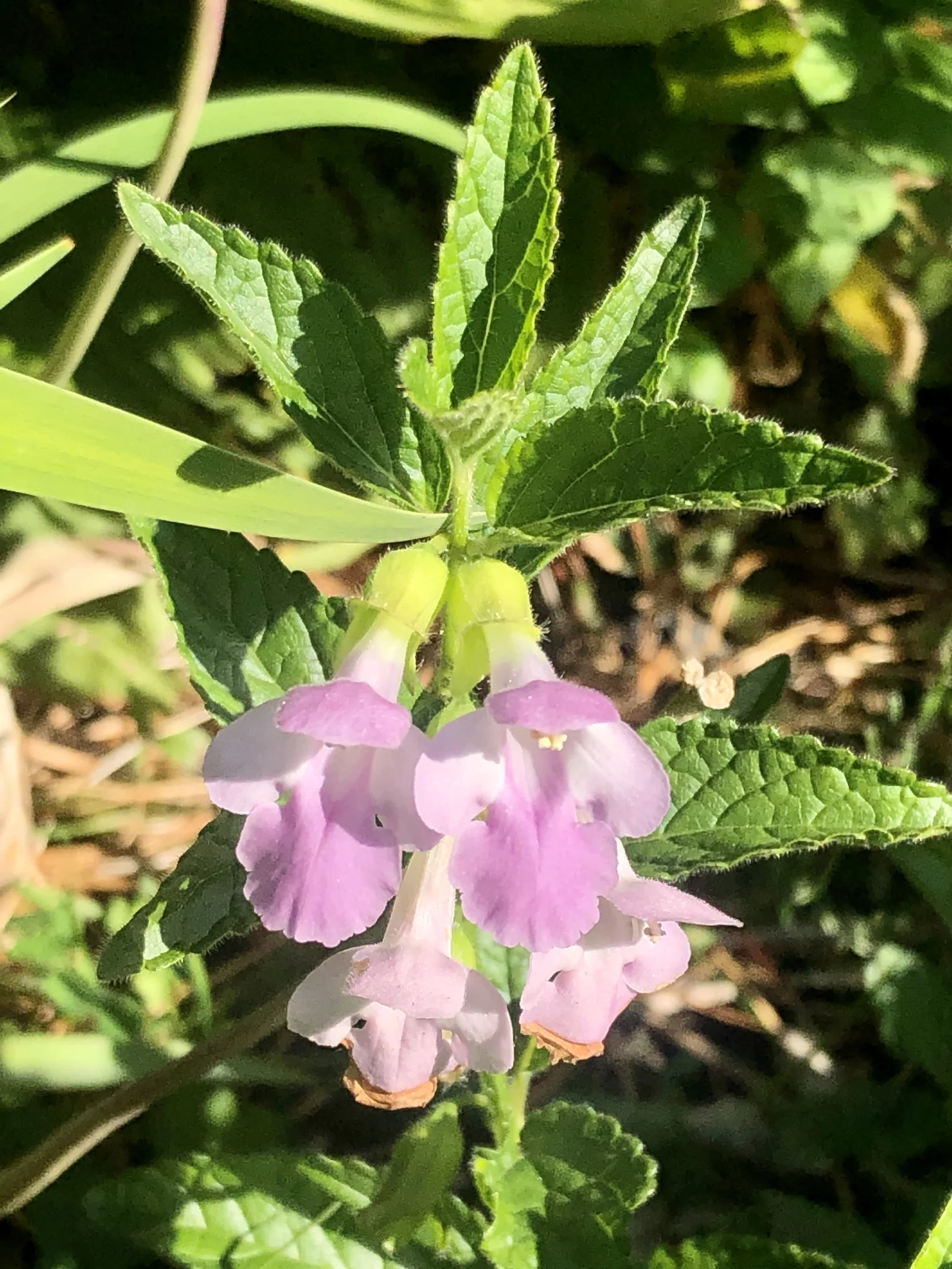
Photograph of *Melittis melissophyllum* by Maarten Christenhusz.

## Methods

### Sample acquisition, flow cytometry and DNA barcoding

A specimen of *M. melissophyllum* (specimen ID KDTOL10690, ToLID daMelMeli1) was used for genome sequencing. It was collected from Royal Botanic Gardens Kew, Richmond, UK (latitude 51.4825, longitude −0.2876) on 2022-09-29. The specimen was collected by Maarten Christenhusz and Sahr Mian and identified by Maarten Christenhusz. The herbarium voucher for the sequenced plant (collector number MC9455) is deposited in the Herbarium, Royal Botanic Gardens, Kew (K), as specimen K001527052.

The genome size was estimated by flow cytometry following the ‘one-step’ method outlined in Pellicer *et al*. (2021) and using propidium iodide as the fluorochrome. The General Purpose Buffer (GPB) supplemented with 3% PVP was used for isolation of nuclei ( Loureiro *et al.*, 2007), and the internal calibration standard was *Solanum lycopersicum* L. ‘Stupické polní rané’ with an assumed 1C-value of 968 Mb ( Doležel *et al.*, 2007).

The initial identification was verified by an additional DNA barcoding process according to the framework developed by Twyford *et al*. (2024). Part of the plant specimen was preserved in silica gel desiccant ( Chase & Hills, 1991). DNA extracted from the dried plant was amplified by PCR for standard barcode markers, with the amplicons sequenced and compared to public sequence databases including GenBank and the Barcode of Life Database (BOLD) ( Ratnasingham & Hebert, 2007). Following whole genome sequence generation, the relevant DNA barcode region was also used alongside the initial barcoding data for sample tracking at the WSI ( Twyford *et al.* , 2024). The standard operating procedures for Darwin Tree of Life barcoding are available on protocols.io.

### Nucleic acid extraction

High molecular weight (HMW) DNA extraction protocols developed by the Tree of Life Core Laboratory at the Wellcome Sanger Institute (WSI) are available on protocols.io ( Howard *et al.*, 2025). Tissue from the daMelMeli1 sample was weighed and triaged to select the appropriate extraction workflow. HMW DNA was prepared for one PacBio HiFi library, for which 58.0 mg of leaf tissue was homogenised using cryoPREP. HMW DNA was extracted using the Automated Plant MagAttract v2 protocol, and then DNA was sheared into an average fragment size of 12–20 kb following the Megaruptor®3 for LI PacBio protocol. Sheared DNA was purified following the Sanger Tree of Life Fragmented DNA clean up: Manual SPRI protocol.

The concentration of the sheared and purified DNA was assessed with a NanoDrop spectrophotometer and a Qubit Fluorometer using the Qubit dsDNA High Sensitivity Assay kit. Fragment size distribution was evaluated by running the sample on the Femto Pulse system. For this sample, final post-shearing DNA measurements included a Qubit concentration of 9.17 ng/μL, a yield of 430.99 ng, and an average fragment size of 13.9 kb. The 260/280 spectrophotometric ratio was 1.89, and the 260/230 ratio was 1.07. The Genomic Quality Number (GQN) was 7.1.

### PacBio HiFi library preparation and sequencing

Library preparation and sequencing took place in the WSI Scientific Operations core. Library DTOL13700591 was prepared with the SMRTbell Prep Kit 3.0 (Pacific Biosciences) according to the manufacturer’s instructions. The kit includes reagents for end repair/A-tailing, adapter ligation, post-ligation SMRTbell bead clean-up, and nuclease treatment. Size selection and clean-up were performed using diluted AMPure PB beads (Pacific Biosciences). DNA concentration was quantified with a Qubit Fluorometer v4.0 (Thermo Fisher Scientific) and the Qubit 1X dsDNA HS assay kit. Final library fragment size was assessed with the Agilent Femto Pulse Automated Pulsed Field CE Instrument (Agilent Technologies) using the gDNA 55 kb BAC analysis kit.

The library was sequenced using the Sequel IIe system (Pacific Biosciences, California, USA). The concentration of the library loaded onto the Sequel system was in the range 40–135 pM. SMRT Link software, a PacBio web-based workflow manager, was used to set up and monitor the run, and to perform primary and secondary analysis of the data upon completion.

### Hi-C

***Sample preparation and crosslinking***

Hi-C data were generated from the daMelMeli1 leaf sample using the Arima-HiC v2 kit (Arima Genomics). Tissue was finely ground using the Covaris cryoPREP Dry Pulverizer (Covaris), and then subjected to nuclei isolation. Nuclei were isolated using a modified protocol based on the Qiagen QProteome Cell Compartment Kit (Qiagen), in which only the Lysis and CE2 buffers were used, with QIAshredder spin columns. After isolation, nuclei were fixed using formaldehyde to a final concentration of 2% to crosslink the DNA. The crosslinked DNA was then digested and biotinylated according to the manufacturer’s instructions. A clean-up step was performed with SPRIselect beads before library preparation. DNA concentration was quantified using the Qubit Fluorometer v4.0 (Thermo Fisher Scientific) and the Qubit HS Assay Kit, following the manufacturer’s instructions.

***Hi-C library preparation and sequencing***

Biotinylated DNA constructs were fragmented using a Covaris E220 sonicator and size-selected to 400–600 bp using SPRISelect beads. DNA was enriched with Arima-HiC v2 kit Enrichment beads. End repair, A-tailing, and adapter ligation were carried out with the NEBNext Ultra II DNA Library Prep Kit (New England Biolabs), following a modified protocol where library preparation occurs while DNA remains bound to the Enrichment beads. Libraries were amplified with KAPA HiFi HotStart mix and a custom Unique Dual Index (UDI) barcode set (Integrated DNA Technologies). Depending on sample concentration and biotinylation percentage determined at the crosslinking stage, libraries were amplified with 10–16 PCR cycles. Post-PCR clean-up was performed with SPRISelect beads. Libraries were quantified using the AccuClear Ultra High Sensitivity dsDNA Standards Assay Kit (Biotium) and a FLUOstar Omega plate reader (BMG Labtech).

Prior to sequencing, libraries were normalised to 10 ng/μL. Normalised libraries were quantified again to create equimolar and/or weighted 2.8 nM pools. Pool concentrations were checked using the Agilent 4200 TapeStation (Agilent) with High Sensitivity D500 reagents before sequencing. Libraries were sequenced on the Illumina NovaSeq 6000, generating paired-end 150-bp reads.

### Genome assembly

Prior to assembly of the PacBio HiFi reads, a database of *k*-mer counts ( *k* = 31) was generated from the filtered reads using FastK. The *k*-mer frequency distribution was analysed with GenomeScope (version 2.1.0) ( Ranallo-Benavidez *et al.*, 2020), providing fitted point estimates of genome size, heterozygosity and repeat content.

The HiFi reads were assembled using Hifiasm ( Cheng *et al.*, 2021) with the --primary option. The Hi-C reads ( Rao *et al.* , 2014) were mapped to contigs using bwa-mem2 ( Vasimuddin *et al.*, 2019), and the contigs were scaffolded in YaHS ( Zhou *et al.*, 2023) with the --break option for handling potential misassemblies. The scaffolded assemblies were evaluated using Gfastats ( Formenti *et al.* , 2022), BUSCO ( Manni *et al.*, 2021) and MerquryFK ( Rhie *et al.*, 2020).

The organelle genomes were assembled using OATK ( Zhou *et al.*, 2025). The comparator for the plastid assembly was the chloroplast genome of *Stachys affinis* (NC_062803.1).

### Assembly curation

The assembly was decontaminated using the Assembly Screen for Cobionts and Contaminants ( ASCC) pipeline. The flat files and maps for curation were generated with TreeVal. Manual curation was conducted primarily in PretextView and HiGlass ( Kerpedjiev *et al.* , 2018). Scaffolds were visually inspected and corrected as described by Howe *et al*. (2021). Manual corrections included 22 breaks, 29 joins, and removal of eight haplotypic duplications. This reduced the scaffold count by 94.9%, reduced the scaffold N50 by 21.9%, and reduced the total assembly length by 16.8%. The curation process is described at sanger-tol/curation-resources. A Hi-C contact map of the primary assembly was generated with PretextSnapshot.

### Assembly quality assessment

The MerquryFK tool ( Rhie *et al.*, 2020) was run in a Singularity container ( Kurtzer *et al.*, 2017) to evaluate *k*-mer completeness and assembly quality for the primary and alternate haplotypes using the *k*-mer database ( *k* = 31) computed prior to genome assembly. The analysis outputs included assembly QV scores and completeness statistics.

The primary assembly was assessed using BlobToolKit ( Challis *et al.*, 2020). PacBio reads were mapped to the primary assembly to derive sequence coverage, and BUSCO ( Manni *et al.*, 2021) was used to assess gene-set completeness. DIAMOND BLASTp searches of extracted BUSCO proteins and DIAMOND BLASTx searches of assembly sequences against UniProt Reference Proteomes ( Buchfink *et al.*, 2021), followed by BLASTn searches of sequences without DIAMOND BLASTx hits against the NCBI nt database ( Altschul *et al.*, 1990), supported taxonomic assignment. Coverage, sequence composition, taxonomic assignment and BUSCO results were integrated into a BlobDir for interactive inspection and visualisation. The snail and blob plots were rendered locally from this hosted BlobDir using BlobTk v0.8.3. The scaffold-length radial scale function for the snail plot was set to linear ( Challis & Blaxter, 2026). The blob plot used circular markers with area proportional to scaffold length ( Challis *et al.*, 2020).

## Genome sequence report

### Sequence data

The genome of a specimen of *M. melissophyllum* was sequenced using Pacific Biosciences single-molecule HiFi long reads, generating 26.81 Gb (gigabases) from 2.78 million reads. We assembled the genome from these reads. GenomeScope analysis using a *p* = 2 model gave a *k*-mer-based haploid genome size estimate of 542.49 Mb, with heterozygosity of 0.22% and repeat content of 54.04%; the full-model fit was 97.70% (Figure 2). Using flow cytometry, the genome size (1C‐value) of the sample was estimated to be 0.58 pg, equivalent to 570.00 Mb. These estimates guided expectations for the assembly. The sequencing data provided approximately 46× coverage of the primary assembly. Hi-C sequencing produced 87.91 Gb from 291.10 million read pairs, which were used to scaffold the primary assembly. Table 1 summarises the specimen and sequencing details.

**Figure 2.**
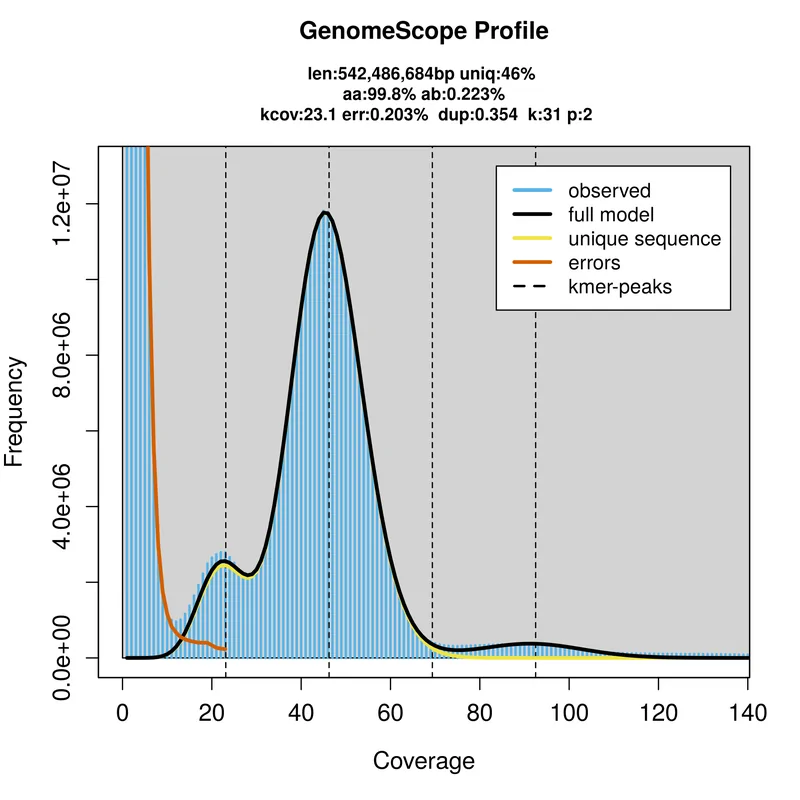
GenomeScope model fitted to the *k*-mer frequency distribution. The plot shows the observed and modelled *k*-mer spectra used to estimate genome size, heterozygosity and repeat content from unassembled sequencing reads.

**Table 1.** Specimen and sequencing data for *M. melissophyllum* (BioProject PRJEB65658)

**Platform**	**PacBio HiFi**	**Hi-C**
**ToLID**	daMelMeli1	daMelMeli1
**Specimen ID**	KDTOL10690	KDTOL10690
**BioSample (source individual)**	SAMEA112290316	SAMEA112290316
**BioSample (tissue)**	SAMEA112290391	SAMEA112290391
**Tissue**	leaf	leaf
**Instrument**	Sequel IIe (1 run)	Illumina NovaSeq 6000
**Library ID & run accession(s)**	DTOL13700591: ERR12015727	ERR12035228
**Read count total**	2.78 million reads	291.10 million read pairs
**Base count total**	26.81 Gb	87.91 Gb

### Assembly statistics

The primary assembly was generated and an alternate haplotype assembly was also deposited at scaffold level in INSDC databases. The primary assembly is proposed as the reference assembly for this species. It has a total length of 436.70 Mb in 99 scaffolds (excluding organelle sequences), with 138 gaps, and a scaffold N50 of 25.55 Mb (Table 2). The primary assembly has a scaffold auN of 27.0 Mb.

**Table 2.** Genome assembly statistics for *M. melissophyllum*

**Genome assembly**	**Primary assembly**	**Alternate haplotype assembly**
**Assembly name**	daMelMeli1.1	daMelMeli1.1 alternate haplotype
**Assembly accession**	GCA_965112285.1	GCA_965112265.1
**Assembly level**	chromosome	scaffold
**Span (Mb)**	436.70	263.16
**Number of chromosomes**	17	-
**Number of contigs**	237	4,470
**Contig N50 (Mb)**	4.48	0.14
**Number of scaffolds (excluding organelle sequences)**	99	4,469
**Scaffold N50 (Mb)**	25.55	0.14
**Scaffold auN (Mb)**	27.0	-
**Longest scaffold length (Mb)**	40.54	6.61
**Organelles**	Mitochondrial genome: 366.69 kb; Plastid genome: 153.23 kb	-

Most of the primary assembly sequence (97.71%) was assigned to 17 chromosome-level scaffolds. These chromosome-level scaffolds, confirmed by Hi-C data, are named according to size (Figure 3; Table 3).

The mitochondrial genome (length 366.69 kb, OZ218192.1) and plastid genome (length 153.23 kb, OZ218193.1) were also assembled. These sequences are included as contigs in the multifasta file of the primary genome submission and as standalone records.

**Figure 3.**
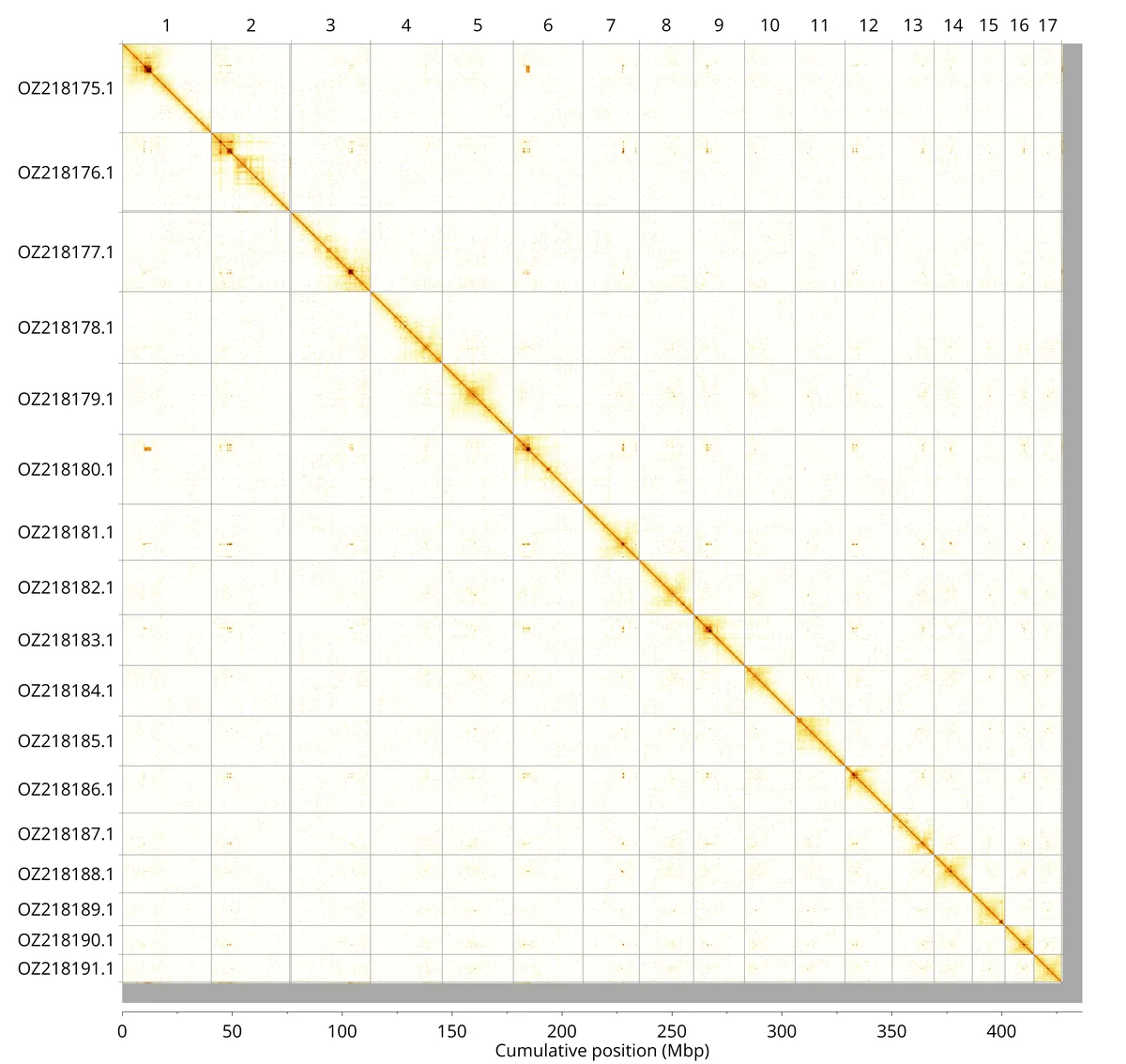
Hi-C contact map of the *M. melissophyllum* primary assembly. Assembled chromosomes are shown in order of size and labelled along the axes, with a megabase scale shown below. The plot was generated using PretextSnapshot.

**Table 3.** Chromosomal pseudomolecules in the primary genome assembly of *M. melissophyllum* daMelMeli1

**INSDC accession**	**Molecule**	**Length (Mb)**	**GC%**
OZ218175.1	1	40.54	36
OZ218176.1	2	36.26	36.50
OZ218177.1	3	35.94	36.50
OZ218178.1	4	32.73	35.50
OZ218179.1	5	32.19	36
OZ218180.1	6	31.67	36
OZ218181.1	7	25.55	36
OZ218182.1	8	24.74	36.50
OZ218183.1	9	23.12	35.50
OZ218184.1	10	22.99	35.50
OZ218185.1	11	22.67	36
OZ218186.1	12	21.37	35
OZ218187.1	13	19.08	36
OZ218188.1	14	17.28	36.50
OZ218189.1	15	14.94	36.50
OZ218190.1	16	13.15	36
OZ218191.1	17	12.50	37.50

### Assembly quality metrics

The combined primary and alternate assemblies achieve an estimated QV of 63.6. The *k*-mer completeness is 92.48% for the primary assembly, 51.48% for the alternate haplotype, and 98.04% for the combined assemblies (Figure 4).

BUSCO v.5.5.0 analysis of the primary assembly using the eudicots_odb10 reference set ( *n* = 2,326) identified 97.5% of the expected gene set (single = 94.1%, duplicated = 3.4%).

**Figure 4.**
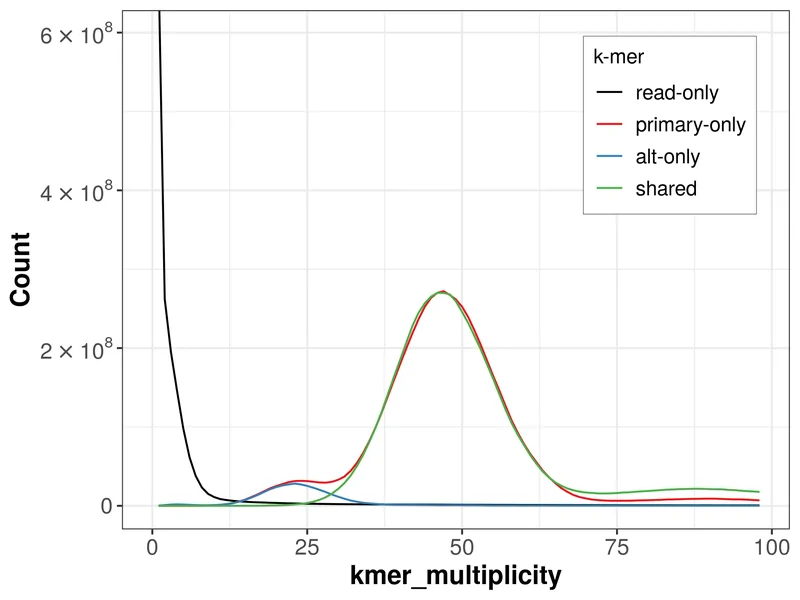
Evaluation of *k*-mer completeness using MerquryFK. This plot illustrates the recovery of *k*‐mers from the original read data in the primary assembly, the alternate haplotype assembly and the combined assemblies. The horizontal axis represents *k*‐mer multiplicity, and the vertical axis shows the number of *k*‐mers. The black curve represents *k*‐mers that appear in the reads but are not assembled, while the remaining curves correspond to *k*‐mers recovered in the assemblies shown.

The snail plot in Figure 5 provides a summary of assembly contiguity, cumulative scaffold count, sequence composition and BUSCO completeness for the primary assembly ( Challis & Blaxter, 2026). The relative sizes of the longest scaffold, scaffold N50 and N90, together with the dark-grey scaffold-length distribution, show how much of the assembly is contained in long scaffolds. The outer composition track shows variation in GC, AT and N content, while the central track shows the cumulative scaffold count and the BUSCO inset summarises gene-set completeness.

The blob plot in Figure 6 displays scaffolds from the primary assembly by GC proportion and base coverage. Marker area is proportional to scaffold length, and colour indicates taxonomic assignment ( Challis *et al.*, 2020).

**Figure 5.**
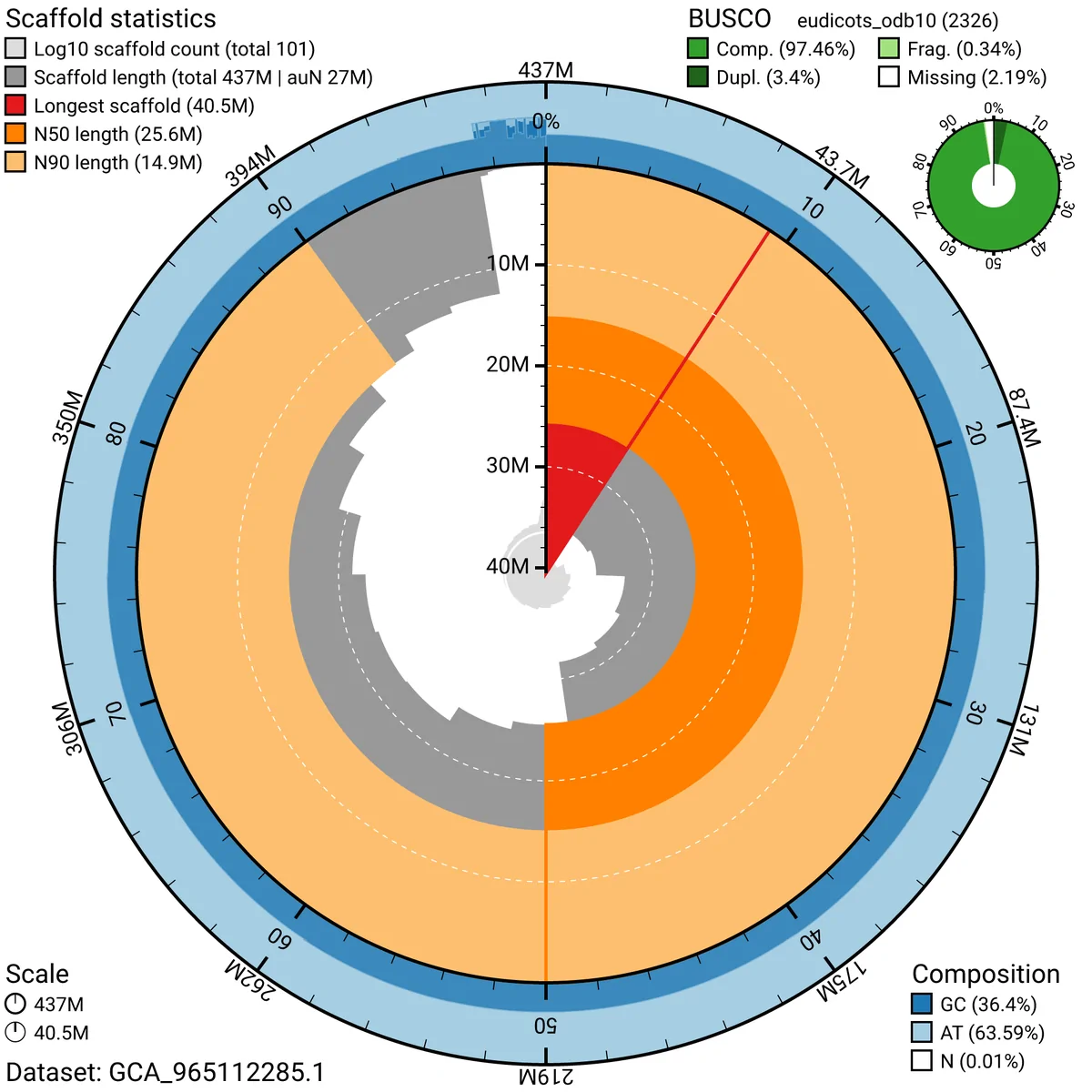
Assembly metrics for daMelMeli1.1, the primary assembly. The BlobToolKit snail plot provides an overview of assembly metrics and BUSCO gene completeness. The circumference represents the length of the primary assembly, and the main plot is divided into 1,000 bins around the circumference. The outermost blue tracks display the distribution of GC, AT, and N percentages across the bins. Scaffolds are arranged clockwise from longest to shortest and are depicted in dark grey. Scaffold length is shown on a linear radial scale, scaled to the longest scaffold. Red shading indicates the longest scaffold, and the deeper orange and pale orange shading represent the N50 and N90 lengths. The summary statistics include scaffold auN, the area under the scaffold Nx curve. The light grey spiral at the centre shows the cumulative scaffold count on a logarithmic scale. A summary of complete, fragmented, duplicated, and missing BUSCO genes in the eudicots_odb10 set is presented at the top right. The assembly can be explored interactively in the BlobToolKit viewer.

**Figure 6.**
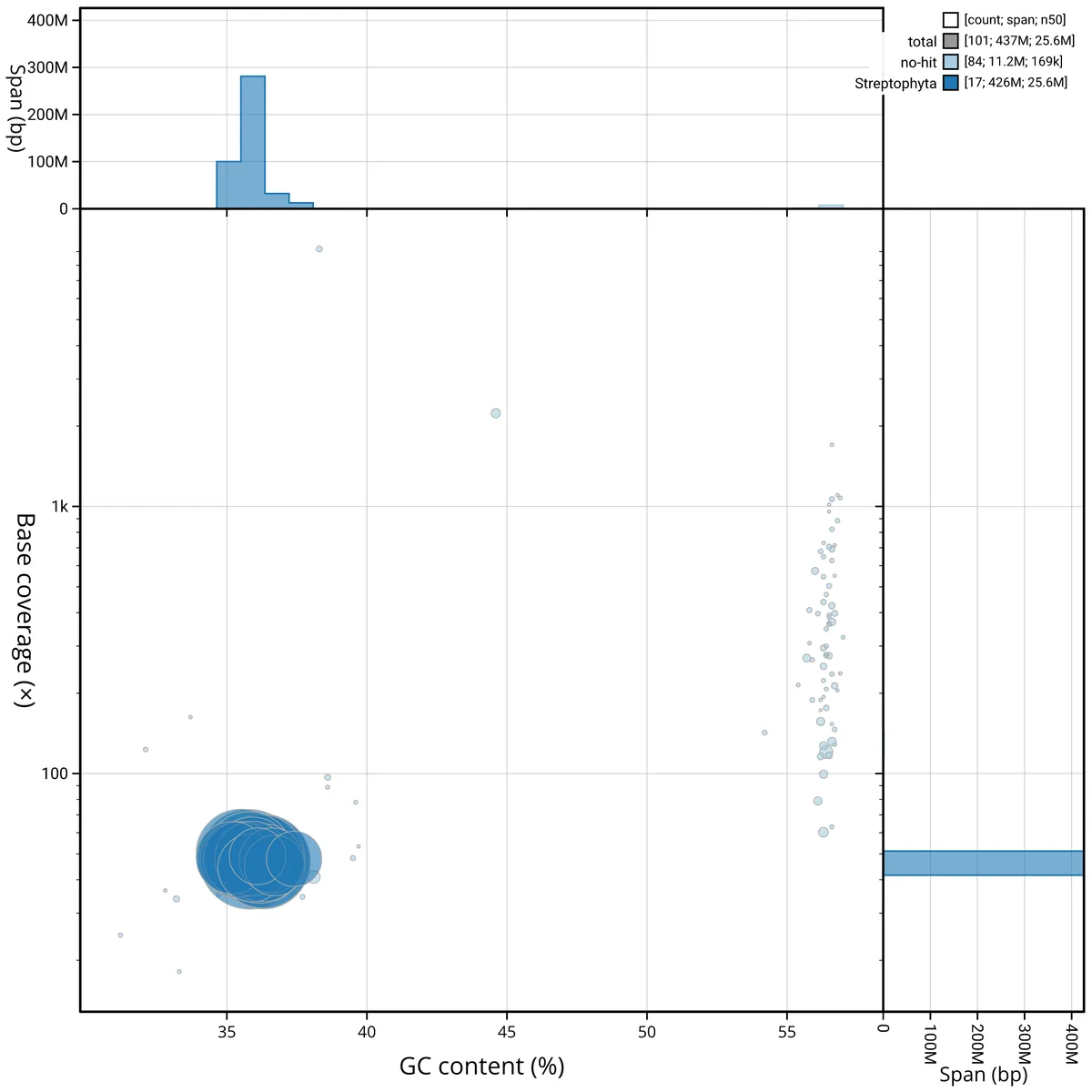
BlobToolKit blob plot for daMelMeli1.1. The plot shows base coverage (vertical axis) and GC content (horizontal axis). Circles represent scaffolds; marker area is proportional to scaffold length, and colour indicates taxonomic assignment. The histograms along the axes display the total length of sequences distributed across different levels of coverage and GC content. The assembly can be explored interactively in the BlobToolKit viewer.

Table 4 lists the assembly metric benchmarks adapted from Rhie *et al.*. (2021) and the Earth BioGenome Project (EBP) Report on Assembly Standards. The three-part EBP metric calculated for the primary assembly is **6.C.Q66**.

**Table 4.** Earth BioGenome Project summary metrics for the *M. melissophyllum* assembly

**Measure**	**Value**	***Benchmark***
EBP summary (primary)	6.C.Q66	*6.C.Q40*
Contig N50 length	4.48 Mb	*> 1 Mb*
Scaffold N50 length	25.55 Mb	*= chromosome N50*
Consensus quality (QV)	Primary: 66.3; alternate: 61.1; combined: 63.6	*≥ 40*
*k*-mer completeness	Primary: 92.48%; alternate: 51.48%; combined: 98.04%	*> 90%*
BUSCO	C:97.5% [S:94.1%, D:3.4%], F:0.3%, M:2.2%, n:2,326	*S > 90%; D < 5%*
Percentage of assembly assigned to chromosomes	97.71%	*≥ 90%*

**Notes:** The EBP summary uses log10(Contig N50); chromosome-level (C) or log10(Scaffold N50); Q (Merqury QV). BUSCO v5.5.0 using the eudicots_odb10 lineage dataset: C=complete; S=single-copy; D=duplicated; F=fragmented; M=missing; n=orthologues.

### Data availability

European Nucleotide Archive: Melittis melissophyllum. Accession number PRJEB65658; https://identifiers.org/ena.embl/PRJEB65658. The genome sequence is released openly for reuse. The *M. melissophyllum* genome sequencing initiative is part of the Darwin Tree of Life Project ( PRJEB40665) and the Sanger Institute Tree of Life Programme ( PRJEB43745). All raw sequence data and the primary and alternate assemblies have been deposited in INSDC databases. The genome will be annotated using available RNA-Seq data and presented through the Ensembl pipeline at the European Bioinformatics Institute. Raw data and assembly accession identifiers are reported in Table 1 and Table 2.

Pipelines used for genome assembly at the WSI Tree of Life are available at https://pipelines.tol.sanger.ac.uk/pipelines. Table 5 lists software versions used in this study.

**Table 5.** Software versions and sources used for *M. melissophyllum*

**Software**	**Version**	**Source**
BLAST	2.14.0	https://ftp.ncbi.nlm.nih.gov/blast/executables/blast+/
BlobToolKit	4.3.9	https://github.com/genomehubs/blobtoolkit
BlobTk (hosted BlobDir)	0.5.1	https://github.com/genomehubs/blobtk
BlobTk (snail and blob plot renderer)	0.8.3	https://github.com/genomehubs/blobtk
BUSCO	5.5.0	https://gitlab.com/ezlab/busco
bwa-mem2	2.2.1	https://github.com/bwa-mem2/bwa-mem2
DIAMOND	2.1.8	https://github.com/bbuchfink/diamond
fasta_windows	0.2.4	https://github.com/tolkit/fasta_windows
FastK	1.2	https://github.com/thegenemyers/FASTK
GenomeScope	2.1.0	https://github.com/tbenavi1/genomescope2.0
Gfastats	1.3.6	https://github.com/vgl-hub/gfastats
Hifiasm	0.19.5-r587	https://github.com/chhylp123/hifiasm
HiGlass	1.13.4	https://github.com/higlass/higlass
MerquryFK	1.1.0-c1	https://github.com/thegenemyers/MERQURY.FK
Minimap2	2.24-r1122	https://github.com/lh3/minimap2
Oatk	0.2	https://github.com/c-zhou/oatk
MultiQC	1.14; 1.17 and 1.18	https://github.com/MultiQC/MultiQC
Nextflow	23.10.0	https://github.com/nextflow-io/nextflow
PretextSnapshot	0.0.6	https://github.com/sanger-tol/PretextSnapshot
PretextView	1.0.3	https://github.com/sanger-tol/PretextView
samtools	1.19.2	https://github.com/samtools/samtools
sanger-tol/ascc	0.1.0	https://github.com/sanger-tol/ascc
sanger-tol/blobtoolkit	0.6.0	https://github.com/sanger-tol/blobtoolkit
sanger-tol/curationpretext	1.4.2	https://github.com/sanger-tol/curationpretext
Seqtk	1.4-r122	https://github.com/lh3/seqtk
Singularity	3.9.0	https://github.com/sylabs/singularity
TreeVal	1.4.0	https://github.com/sanger-tol/treeval
YaHS	1.1a.2	https://github.com/c-zhou/yahs

### Author information

- Members of the Royal Botanic Gardens Kew Genome Acquisition Lab
- Members of the Plant Genome Sizing Collective
- Members of the Darwin Tree of Life Barcoding collective
- Members of the Wellcome Sanger Institute Tree of Life Management, Samples and Laboratory team
- Members of Wellcome Sanger Institute Scientific Operations – Sequencing Operations
- Members of the Wellcome Sanger Institute Tree of Life Core Informatics team
- Members of the Tree of Life Core Informatics collective
- Members of the Darwin Tree of Life Consortium

### Wellcome Sanger Institute – Legal and Governance

The materials that have contributed to this genome note have been supplied by a Darwin Tree of Life Partner. The submission of materials by a Darwin Tree of Life Partner is subject to the **‘Darwin Tree of Life Project Sampling Code of Practice’**, which can be found in full on the Darwin Tree of Life website. By agreeing with and signing up to the Sampling Code of Practice, the Darwin Tree of Life Partner agrees they will meet the legal and ethical requirements and standards set out within this document in respect of all samples acquired for, and supplied to, the Darwin Tree of Life Project. Further, the Wellcome Sanger Institute employs a process whereby due diligence is carried out proportionate to the nature of the materials themselves, and the circumstances under which they have been/are to be collected and provided for use. The purpose of this is to address and mitigate any potential legal and/or ethical implications of receipt and use of the materials as part of the research project, and to ensure that in doing so we align with best practice wherever possible. The overarching areas of consideration are:

- Ethical review of provenance and sourcing of the material
- Legality of collection, transfer and use (national and international)

Each transfer of samples is further undertaken according to a Research Collaboration Agreement or Material Transfer Agreement entered into by the Darwin Tree of Life Partner, Genome Research Limited (operating as the Wellcome Sanger Institute), and in some circumstances, other Darwin Tree of Life collaborators.
