# Supplementary material for: The genome sequence of Bastard Balm, Melittis melissophyllum L. (Lamiales: Lamiaceae)
Source: Wellcome Open Res. 2026 Sep 18;11:702. [Version 1] doi: 10.12688/wellcomeopenres.27850.1 (PMC13654072; doi:10.12688/wellcomeopenres.27850.1)
Supplement: Peer review history

# Reviewer report: Reviewer response for version 1

**DOI:** 10.21956/wellcomeopenres.30647.r171212

**Published:** 2026-10-10

**Authors:** Benjamin R. Lichman, Samuel J. Smit

**Affiliations:** 1 University of York, York, England, UK

The authors assembled a chromosome-level genome for *Melittis melissophyllum*, a monotypic genus within the Lamiaceae. This is a valuable addition to the ever expanding genomic resources for this family. All metrics evaluated indicate that the primary assembly is of high quality and complete. The methods are clearly described and the data is publicly accessible.

Are the datasets clearly presented in a usable and accessible format, and the assembly and annotation available in an appropriate subject-specific repository?

Yes

Are sufficient details of the sequencing and extraction, software used, and materials provided to allow replication by others?

Yes

Are the rationale for sequencing the genome and the species significance clearly described?

Yes

Are the protocols appropriate and is the work technically sound?

Yes

Reviewer Expertise:

Mint family, specialised metabolism, biosynthesis, plant genomics

We confirm that we have read this submission and believe that we have an appropriate level of expertise to confirm that it is of an acceptable scientific standard.

# Reviewer report: Reviewer response for version 1

**Published:** 2026-09-18

**Authors:** Automated Benchmarking

**Affiliations:** 1 Wellcome Sanger Institute, Hinxton, UK

As this article is a Genome Note, Automated Benchmarking has been used to determine whether the reported assembly passes four defined checks established by the Earth BioGenome Project: the raw and assembled data are publicly available, the Earth BioGenome Project three-part metric is met, the k-mer completeness exceeds 90%, and the BUSCO score passes the stated threshold. For Melittis melissophyllum (daMelMeli1), all four checks passed. Data available: pass. ENA study PRJEB65658, raw-read accessions ERR12015727 and ERR12035228, and assembly accessions GCA_965112285.1 and GCA_965112265.1 are public. Earth BioGenome Project three-part metric: pass. The primary assembly is reported as 6.C.Q66, meeting the 6.C.Q40 reference standard. k-mer completeness: pass. The combined haplotype assemblies have 98.04% k-mer completeness, exceeding the Earth BioGenome Project target of 90%. BUSCO: pass. The primary assembly, which is proposed as the reference assembly for this species, has 97.5% complete BUSCOs (94.1% single-copy and 3.4% duplicated). BUSCO software version: 5.5.0; lineage dataset: eudicots_odb10. It meets the plant-specific target of greater than 90% complete BUSCOs. This decision uses values reported in the Genome Note and public accession records.

Reviewer Expertise:

NA

I confirm that I have read this submission and believe that I have an appropriate level of expertise to confirm that it is of an acceptable scientific standard.
